# Influence of Formulation Parameters on Redispersibility of Naproxen Nanoparticles from Granules Produced in a Fluidized Bed Process

**DOI:** 10.3390/pharmaceutics12040363

**Published:** 2020-04-16

**Authors:** Martin Wewers, Stefan Czyz, Jan Henrik Finke, Edgar John, Bernard Van Eerdenbrugh, Michael Juhnke, Heike Bunjes, Arno Kwade

**Affiliations:** 1 Institute for Particle Technology, Technische Universität Braunschweig, Volkmaroder Str. 5, 38104 Braunschweig, Germany; jan.finke@tu-braunschweig.de (J.H.F.); a.kwade@tu-braunschweig.de (A.K.); 2 Center of Pharmaceutical Engineering (PVZ), Technische Universität Braunschweig, 38106 Braunschweig, Germany; s.czyz@tu-braunschweig.de (S.C.); heike.bunjes@tu-braunschweig.de (H.B.); 3 Institute of Pharmaceutical Technology, Technische Universität Braunschweig, Mendelssohnstr. 1, 38106 Braunschweig, Germany; 4 Novartis Pharma AG, 4002 Basel, Switzerland; edgar.john@novartis.com (E.J.); bernard.van_eerdenbrugh@novartis.com (B.V.E.); michael.juhnke@novartis.com (M.J.)

**Keywords:** wet media milling, poor aqueous solubility, naproxen, API nanosuspension, fluidized bed granulation, formulation parameters, reconstitution

## Abstract

The particle size reduction of active pharmaceutical ingredients is an efficient method to overcome challenges associated with a poor aqueous solubility. With respect to stability and patient’s convenience, the corresponding nanosuspensions are often further processed to solid dosage forms. In this regard, the influence of several formulation parameters (i.e., type of carrier material, type and amount of additional polymeric drying excipient in the nanosuspension) on the redispersibility of naproxen nanoparticle-loaded granules produced in a fluidized bed process was investigated. The dissolution rate of the carrier material (i.e., sucrose, mannitol, or lactose) was identified as a relevant material property, with higher dissolution rates (sucrose > mannitol > lactose) resulting in better redispersibility of the products. Additionally, the redispersibility of the product granules was observed to improve with increasing amounts of polymeric drying excipient in the nanosuspension. The redispersibility was observed to qualitatively correlate with the degree of nanoparticle embedding on the surface of the corresponding granules. This embedding was assumed to be either caused by a partial dissolution and subsequent resolidification of the carrier surface dependent on the dissolution rate of the carrier material or by resolidification of the dissolved polymeric drying excipient upon drying. As the correlation between the redispersibility and the morphology of the corresponding granules was observed for all investigated formulation parameters, it may be assumed that the redispersibility of the nanoparticles is determined by their distance in the dried state.

## 1. Introduction

Novel potential drug candidates often exhibit a poor aqueous solubility [1,2]. Due to the fact that this potentially compromises the bioavailability, several formulation options (e.g., salt formation, complexation with cyclodextrins, and amorphization) have been developed in order to overcome issues associated with poor aqueous solubility [3]. One specific option to address this is the reduction of the particle size into the submicron size range. The underlying benefit of this approach is believed to be mainly driven by the increase in dissolution rate, as the latter is directly proportional to the surface area of a compound according to the Noyes–Whitney/Nernst–Brunner equation [4,5,6].

Generally, nanosuspensions of active pharmaceutical ingredients (APIs) can be produced either by bottom-up or top-down processes. During a bottom-up process, nanoparticles are formed by precipitation of solutions. While this offers the possibility to generate amorphous nanoparticles [7,8], downsides of this approach are that corresponding processes are often difficult to control, yield low API concentrations, and result in related products that are challenging regarding stability and disintegration of final dosage forms. As a result, bottom-up processes are less commonly applied compared to so-called top-down processes [9]. Using the latter, nanoparticles are generated by mechanical particle size reduction of coarse APIs. In this regard, wet media milling is the most frequently used top-down method for the production of nanosuspensions in the pharmaceutical industry [9] and has been intensively studied [10,11,12,13,14,15,16,17].

Irrespective of the route of production, API nanosuspensions present several issues concerning their stability. Apart from potential chemical degradation and microbial contamination, nanosuspensions are especially prone to physical stability issues. As the increase in surface area by comminution also leads to an increase in the Gibbs free energy, nanosuspensions are thermodynamically unstable systems tending to reduce their energy by agglomeration. Furthermore, Ostwald ripening may occur, especially when nanosuspensions with broad particle size distributions are produced, as larger particles may grow at the expense of smaller ones. These physical instabilities may result in an alteration of the particle size in the suspensions and consequently in a deterioration of dissolution properties and finally bioavailability [18]. In order to overcome these stability issues and/or to enhance patient convenience, API nanosuspensions are often converted into solid products by suitable drying methods. To ensure proper redispersibility of the dry products, matrix formers are often applied in order to prevent nanoparticle agglomeration during drying [19,20,21,22,23].

Generally, solidification of API nanosuspensions can be achieved by well-established unit operations. Spray-drying [7,21,24,25] and freeze-drying [20,26,27] are often described. However, these processes have certain disadvantages concerning heat exposure and efficiency and/or often yield products with compromised properties (e.g., flowability, particle size, hygroscopicity) towards subsequent processing into a final dosage form.

Alternatively, API nanosuspensions can be processed in fluidized bed devices. Although the corresponding processes normally yield products with superior properties towards subsequent processing, e.g., into tablets [28], the number of publications regarding their applicability for conversion of API nanosuspensions into solid products is rather low. Available literature describes bead-coating/layering with API nanosuspensions [29,30,31] or the application of the corresponding suspensions as granulation liquids in fluidized granulation processes [32,33,34,35,36]. Most of these studies focus on the general feasibility of applying API nanosuspensions in the corresponding processes by showing enhanced dissolution profiles for nanoparticle-loaded products compared to the coarse API particles [29,30,31,32,33,34,35,36]. However, the degree of nanoparticle agglomeration during drying, typically assessed by redispersibility studies of the dry product, was investigated only in a few studies, although it has been shown to be an important criterion to assess the overall performance of the dried nanosuspension, directly influencing the dissolution rate and/or bioavailability [32,33,34]. As a result, there is still a lack of systematic understanding of the relationship between formulation and/or process parameters and performance of the resulting product when using a fluidized bed process.

In this study, a nanosuspension containing naproxen was applied as a granulation liquid during fluidized bed granulation. The influence of the applied formulation parameters on the redispersibility of the resulting granules was of particular interest. Formulation parameters (i.e., carrier particle type, amount and type of polymeric excipient in the nanosuspension) were systematically varied in order to evaluate the relationship with the extent of nanoparticle agglomeration during drying. The product performance (i.e., redispersibility) was related to the structure of the corresponding granules, enabling a better understanding of the application of API nanosuspensions in a fluidized granulation process.

## 2. Materials and Methods

### 2.1. Materials

In this study, micronized naproxen (kindly provided by Novartis Pharma AG, Switzerland) was used as a poorly water-soluble model API. During milling, the naproxen particles were electrosterically stabilized against agglomeration by a combination of either vinylpyrrolidone–vinyl acetate copolymer (PVP/VA, Kollidon VA 64, gift from BASF SE, Germany) or hydroxypropyl methyl cellulose (HPMC, Pharmacoat 603, gift from Shin-Etsu Chemical, Japan) and sodium dodecyl sulfate (SDS, Sigma Aldrich, USA). The stabilizers PVP/VA and HPMC were also used as additional drying excipient/matrix formers during fluidized bed granulation.

Sucrose (Nordzucker AG, Germany), mannitol (Pearlitol 160 C, Roquette Frères, France) and monohydrous lactose (lactose, Granulac 140, Meggle AG, Germany) were used as carrier materials in the fluidized bed granulation process. As the applied sucrose showed a significantly different mean particle size in comparison to mannitol and lactose, fines of sucrose were separated by air jet sieving in order to exclude any influence of the carrier particle size on the granulation investigations. Sieving was carried out using an e200 LS air jet sieve (Hosokawa Alpine AG, Germany). Batches of 100 g sucrose were classified for 15 min using a 20 µm sieve at a pressure of 4000 Pa. The procedure was repeated twice, and sieve retention was used for the granulation trials while the separated fines smaller than 20 µm were discarded.

Characteristic properties of the carrier materials can be found in Table 1. Particle sizes and specific surface areas were determined by laser diffraction (see Section 2.4.1) and solid densities *ρ_solid_* were derived from gas pycnometry measurements (see Section 2.4.3).

If applied, water was used in bidistilled quality.

### 2.2. Production of Naproxen Nanosuspensions

Naproxen nanosuspensions were produced in batches of 500 g in a stirred media mill (MiniCer, kind loan of Netzsch Feinmahltechnik GmbH, Germany). The mill was operated in recirculation mode with a constant mass flow of about 35 kg·h^−1^. The stirrer tip speed was set to ν = 9 m·s^−1^, and the temperature of the suspension was kept constant at 25 ± 1 °C at the outlet of the milling chamber. Yttrium-stabilized zirconium oxide (ZrO_2_) grinding media (ρ_GM_ = 6067 kg·m^−3^) with a mean size of X_50,GM_ = 315 µm (Sigmund Lindner GmbH, Germany) were used. The filling ratio of the grinding chamber with the grinding media was set at ϕ_GM_ = 0.8. Micronized naproxen particles were processed in an aqueous suspension with a mass concentration of *c_m,API_* = 10 wt%. For stabilization, either 2.50 wt% PVP/VA or HPMC in combination with 0.25 wt% SDS was applied. To achieve a sufficiently small particle size, the milling duration was set to 6 h.

### 2.3. Fluidized Bed Granulation

The milled nanosuspensions (*m_NS,orig._* = 50 g of original suspension) were sprayed onto fluidized carrier particles (sucrose, mannitol, lactose) in a fluidized bed device (MiniGlatt, Glatt GmbH, Germany). In case of the application of additional polymer (PVP/VA or HPMC), acting as binder and matrix former to embed API nanoparticles, the corresponding amounts of these polymers were dissolved directly in the nanosuspension prior to spraying it onto the carrier and drying the whole system in the fluidized bed granulation process. As a result, polymer-to-API mass ratios ζ from 0.250 (original nanosuspension) to 0.875 (PVP/VA) or 0.750 (HPMC) were obtained in the product granules. In order to keep the theoretical API content of the granules constant, the carrier particle mass was reduced by subtracting the corresponding amounts of additional polymer (*m_carrier_* = 50 g for original nanosuspension). The corresponding mass of the additional polymer in the suspension *m_polymer,add._*, the corresponding masses of the sprayed granulation liquid *m_GL_*, and the reduced mass of the carrier particles *m_carrier,red._* can be calculated according to Equations (1)–(3), respectively.

(1) $$m_{polymer,add.}=\frac{\left(\zeta -0.25\right)\cdot m_{NS,orig.}}{c_{m,API}}$$

(2) $$m_{GL}=m_{NS,orig.}+m_{polymer,add.}$$

(3) $$m_{carrier,red.}=m_{carrier}-m_{polymer,add.}$$

The resulting mass fractions of the carrier material *c_m,carrier_*, the polymer *c_m,polymer_*, and the API *c_m,API_* in the product granules (neglecting minor contents of SDS) are listed in Table 2.

For all granulation trials, the process parameters were kept constant. The atomizing air pressure was set to 0.7 bar and the nanosuspension was applied with a mean feed rate of approximately 1.5 g·min^−1^. The inlet fluidization air was preheated to 65 °C and process air pressures of 0.12 to 0.30 bar were applied to maintain sufficient fluidization of the carrier particles during the process.

### 2.4. Characterization of Materials and Granules

#### 2.4.1. Particle Size Measurement

Particle size of nanosuspensions (original or redispersed) was measured by means of dynamic light scattering (DLS) with a Zetasizer nano ZSP (Malvern Panalytical Ltd., United Kingdom). Before measurement, samples were adequately diluted with saturated and filtered (0.2 µm pore size) naproxen solution. Diluted samples were then equilibrated for 300 s to ensure proper temperature conditioning during measurements. Subsequently, measurements were performed at 25 °C for 180 s. Each sample was measured in triplicate and the mean values of the z-average (z-avg.) and polydispersity index (PdI) were used as characteristic values for the nanosuspensions. Prior to the measurements, the refractive index (RI) of naproxen was determined by applying Saveyn’s multiple solvent approach [37] at 25 °C and a wavelength of 589.3 nm with an Abbemat MW multiwavelength refractometer (Anton Paar GmbH, Austria). The same device was used for the determination of the RI of the saturated and filtered naproxen solution used as a dilution medium during measurements. The corresponding viscosity was determined in an Ubbelohde viscometer 52,503 (Schott-Geräte GmbH, Germany) at 25 °C. In order to calculate the dynamic viscosity, the density of the dilution medium was determined with a density meter DMA 46 (Anton Paar GmbH, Austria) at 25 °C.

In addition to DLS, particle sizes of redispersed suspensions were also characterized by means of laser diffraction (LD) using a Mastersizer 3000 (Malvern Panalytical Ltd., United Kingdom) with a Hydro MV dispersing unit. This complementary application of different particle size measurement methods provides a more comprehensible insight into particle size distributions as in DLS large agglomerates may sediment, withdrawing themselves from measurements, while LD does not possess as high resolution in the nanometer range as DLS possesses. LD measurements were conducted in saturated and filtered naproxen solution at the lowest possible stirring speed of 500 rpm in order to prevent potential dispersion of nanoparticle agglomerates. Measurements were conducted for 20 s, and each sample was measured in triplicate. Data evaluation was performed applying the Mie theory using the RIs of naproxen and the dispersion medium stated above.

Particle sizes of the raw carrier materials were characterized by means of LD using a Mastersizer 3000 (Malvern Panalytical Ltd., United Kingdom) with an Aero S dispersing unit. Measurements were conducted in triplicate with a dispersing pressure of 2.5 bar. Data evaluation was performed using the Fraunhofer theory. Additionally, data were used to extract the specific surface areas of the carrier materials by taking the solid densities *ρ_solid_* of the materials (see Section 2.1 and Section 2.4.3) into account.

#### 2.4.2. Redispersibility of Granules

For characterizing redispersibility of the product granules, samples were dispersed in saturated and filtered naproxen solution on a magnetic stirrer for 15 min to facilitate complete dissolution of the drying excipients (i.e., carrier materials, polymeric matrix former). The resulting dispersions were measured by means of DLS (see Section 2.4.1), and mean particle size (i.e., z-avg.) and width of the corresponding particle size distribution (i.e., PdI) were recorded. For better comparability of different nanosuspensions, the redispersibility index (RDI) [26] was calculated by normalizing the mean particle size of the redispersed suspension (z-avg._redisp._) to the mean particle size of the original nanosuspension (z-avg._orig._).

(4) $$\mathrm{RDI}=\frac{z\text{-}\mathrm{avg}._{\mathrm{redisp}.}}{z\text{-}\mathrm{avg}._{\mathrm{orig}.}}$$

Furthermore, redispersed suspensions were characterized by means of LD (see Section 2.4.1) to get a deeper understanding of the agglomeration state of the API particles, as particles coarser than about 1 µm cannot be detected reliably by means of DLS. The corresponding value in the cumulative particle size distribution located at the transition of the submicron and the micron size range (i.e., Q_3_(X < 1 µm)) was used as a characteristic value referred to as the redispersed volume fraction (RVF, visualized in Section 3.2.1, Figure 2b). The corresponding value characterizes the proportion of nanoparticles that could be successfully redispersed from the product granules.

#### 2.4.3. Gas Pycnometry

Solid densities *ρ_solid_* of the carrier materials were determined with a helium pycnometer Ultrapyc 1200e (Quantachrome GmbH & Co., Germany).

#### 2.4.4. Apparent Intrinsic Dissolution Rate of Carrier Materials

Apparent intrinsic dissolution rates (IDRs) of the carrier materials were determined according to Ph. Eur. 2.9.29 [38] with slight modifications. For this purpose, compacts of carrier materials were produced with a compaction simulator Styl’One Evolution (MEDEL’PHARM, France) using 11.28 mm flat-faced, round punches. Carrier compacts had a mass *m_compact_* of 200 mg and were compacted under adapted pressure to obtain comparable porosities ε_compact_ calculated according to Equation (5) and listed in Table 3. The variables *h_compact_* and *d* refer to the height and diameter of the carrier compacts, and *ρ_solid_* refers to the solid density of the carrier materials (see Section 2.4.3).

(5) $$\varepsilon_{compact}=1-\frac{m_{compact}}{\pi \cdot {\left(\frac{d}{2}\right)}^{2}\cdot h_{compact}\cdot \rho_{solid}}$$

**Figure 1:**
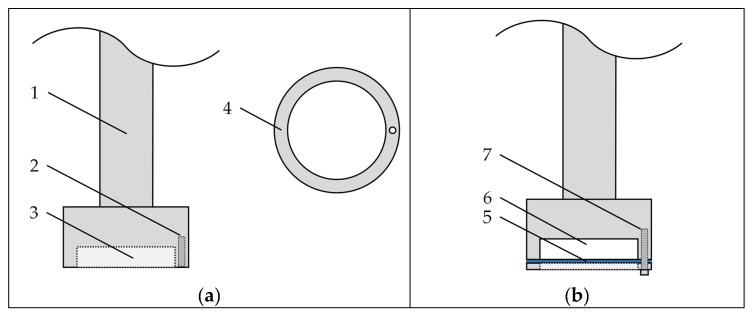
Schematic illustration of the stirring device for determination of the apparent intrinsic dissolution rate. (**a**) Disassembled view with 1. stirring axle, 2. bore for fixation of the cover, 3. cavity for carrier compact, and 4. cover for holding carrier compact in place (top view). (**b**) Assembled view with 5. filter paper, 6. carrier compact, and 7. screw for fixation of cover.

The carrier compact was placed in a special, self-constructed stirring device schematically depicted in Figure 1.

A cellulose filter paper was placed beneath the carrier compact (i.e., at the compact–water interface) in order to prevent rapid disintegration and dispersion of the carrier compact. While the filter paper may have an impact on the dissolution rates obtained, results can be compared between carrier materials, as the experimental setup was identical for all experiments. However, as the influence of the filter paper on the IDR was not evaluated, obtained values will be referred to as the apparent IDRs of the carrier materials. After preparation, the stirring device with the compact was placed in a beaker with tempered water (25 °C) and rotated with a fixed frequency of 100 min^−1^. Samples were taken after given periods of time, and the concentration of dissolved carrier material was determined using an Abbemat MW multiwavelength refractometer (Anton Paar GmbH, Austria).

#### 2.4.5. Scanning Electron Microscopy

Surface morphology of nanoparticle-loaded granules was investigated by scanning electron microscopy (SEM) using a Helios G4 CX (Thermo Fischer Scientific, USA). Powder samples were spread on double-sided adhesive carbon pads and sputtered with platinum (5 nm, EM ACE600, Leica Microsystems, Germany) before investigations. Afterwards, investigations were conducted with an acceleration velocity of 2–4 kV.

#### 2.4.6. Confocal Raman Microscopy

In order to evaluate the thickness of API-containing layers on the product granules, confocal Raman microscopy was chosen, as it is capable of identifying chemical compounds in microscopic dimensions in three-dimensional volumes. It was conducted using an Alpha 300R microscope (WITec GmbH, Germany) with an excitation wavelength of 532 nm, operated at a laser power of approximately 5.4 mW and an integration time of 0.1 s. Depth scans of the granules with a size of 10 µm × 10 µm were performed with a step size of 0.2 µm using 100-fold magnification. The data were postprocessed using cosmic ray removal, noise filtering and background subtraction. Afterwards, datasets were compared with reference data of pure naproxen and carrier materials in order to visualize the naproxen distribution in a Raman chemical map.

## 3. Results and Discussion

### 3.1. Production of Naproxen Nanosuspensions

Wet media milling yielded nanosuspensions of naproxen meeting the quality criteria of a z-avg. below 250 nm and a PdI below 0.2, as defined by Bitterlich et al. [14]. Data in Table 4 (standard deviations were below 2 nm for the z-avg. and 0.01 for the PdI) indicate that both applied stabilizers yielded nanosuspensions with similar particle size properties (i.e., z-avg. and PdI) after 6 h of wet media milling. However, both suspensions show certain instabilities, as indicated by an increase in the particle size and/or the PdI upon storage at ambient temperatures. A fairly rapid increase in particle size could be observed during the first 3 days of storage (approximately 11% for PVP/VA, approximately 8% for HPMC), followed by much less pronounced increases between 3 and 7 days of storage (approximately 5% for PVP/VA, approximately 2% for HPMC). Therefore, subsequent nanosuspension drying experiments were conducted within 3 to 7 days after production of the nanosuspension to exclude any influence of the particle size during the subsequent drying process.

### 3.2. Fluidized Bed Granulation of Naproxen Nanosuspensions

For the fluidized bed granulation experiments, API contents of 8.87 wt% (theoretical) naproxen were targeted in the granules. Although corresponding experiments led to differences in process yield and naproxen content of the granules, these differences were not further investigated, as yields were over 80%, and deviations of obtained versus theoretical API contents were all below 10% (data not shown). As no systematic variations of these properties for the different formulation parameters were observed, differences were attributed to common process and analytical fluctuations. However, the main focus of the study lies on the influence of the investigated formulation parameters on the particle size of naproxen after the drying process (i.e., redispersibility). Although the dissolution rate and content uniformity of the API are important properties for the final characterization of the process, the redispersibility is a preliminary quality indicator for the products and the first step towards finally improved bioavailability. In this regard, it has to be noted that only the steady state redispersibility after a sufficient duration of redispersion (see Section 2.4.2) was investigated, neglecting possible differences in the suspension reformation process. However, these differences were assumed to be marginal based on the similar formulations and steady state particle sizes after redispersion are assumed to give valuable information to fundamentally understand the present process. In this context, it has to be additionally noted that fluidized granulation experiments were conducted only once for each formulation in this study. However, in a preliminary study, the reproducibility concerning the redispersibility of the nanoparticle-loaded granules was investigated and considered to be sufficient. Standard deviations of the characteristics for redispersibility were below 0.05 for the RDI obtained by DLS and below 5% for the RVF obtained by LD for three separately produced batches of nanoparticle-loaded granules. Additionally, redispersibility of granules was observed to be insusceptible to minor fluctuations of process parameters.

#### 3.2.1. Influence of Carrier Material

**Figure 2:**
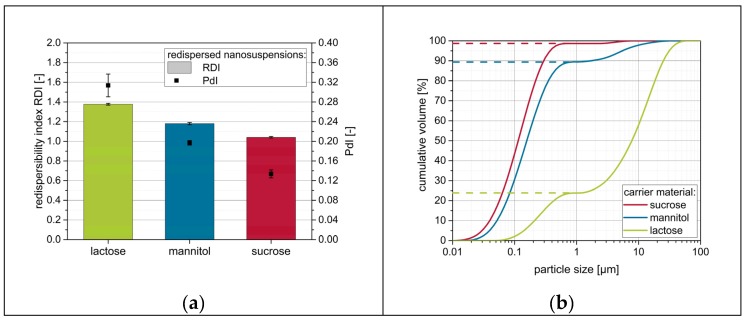
Influence of the carrier particle type on the redispersibility of granules produced with a naproxen nanosuspension without additional drying excipients (except the stabilizing polymer PVP/VA). Measurements were carried out by means of (**a**) DLS and (**b**) LD (dashed lines refer to extracted redispersed volume factions).

In order to investigate the influence of the carrier material on the redispersibility of naproxen nanoparticles from the dry products, nanosuspensions without additional drying excipients (except the stabilizer, i.e., PVP/VA) were applied to fluidized carrier particles. Data obtained from the redispersibility studies are shown in Figure 2.

Data suggest that nanoparticles were at least partially reconstitutable from the granules for all investigated carrier materials, although no further drying excipient/matrix material besides the stabilizers in the nanosuspensions was applied. However, distinct differences can be observed depending on the carrier material used. For lactose as carrier material, redispersibility was rather poor, as indicated by an RDI of 1.37 and an RVF below 0.25. The application of mannitol as a carrier material resulted in a less compromised nanoparticle size with an RDI value of 1.18 and RVF of nearly 0.9. Finally, sucrose showed the best results, with granules that were nearly completely redispersible to the originally applied nanosuspensions as indicated by RDI and RVF of nearly 1.0.

Data additionally reveal that both measurement techniques result in similar conclusions regarding the redispersibility of the samples. An increase in RDI and PdI obtained from DLS measurements always correlates with a reduced RVF extracted from LD measurement data. However, both measurement techniques have their advantages and disadvantages. Measurements by means of DLS offer the possibility to accurately measure the particle size in the submicron range, but particles larger than 1 µm cannot be measured reliably. Furthermore, corresponding particles may sediment during measurements, as measurements are carried out in cuvettes under the avoidance of any convective flow due to the measurement principle. The latter drawback is not relevant during LD measurements that are carried out under constant circulation of the sample through a measurement cell. Furthermore, LD measurements with the present device (see Section 2.4.1) are applicable over a wide size range enabling the concomitant detection of redispersed nanoparticles and micron-sized agglomerates. However, here coarser particles tend to superimpose smaller particles due to their higher scattering intensity. Therefore, the combination of both measurement techniques is deemed necessary for in-depth evaluation of redispersibility.

**Figure 3:**
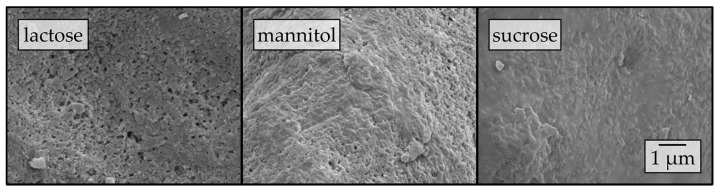
Surface morphologies of granules produced with a naproxen nanosuspension (stabilized with PVP/VA) without additional drying excipients and lactose, mannitol, and sucrose as carrier materials in a fluidized granulation approach, assessed by SEM.

To elucidate the influence of the carrier material on the redispersibility in more detail, surface morphologies of the corresponding granules were investigated by means of SEM (Figure 3).

**Figure 4:**
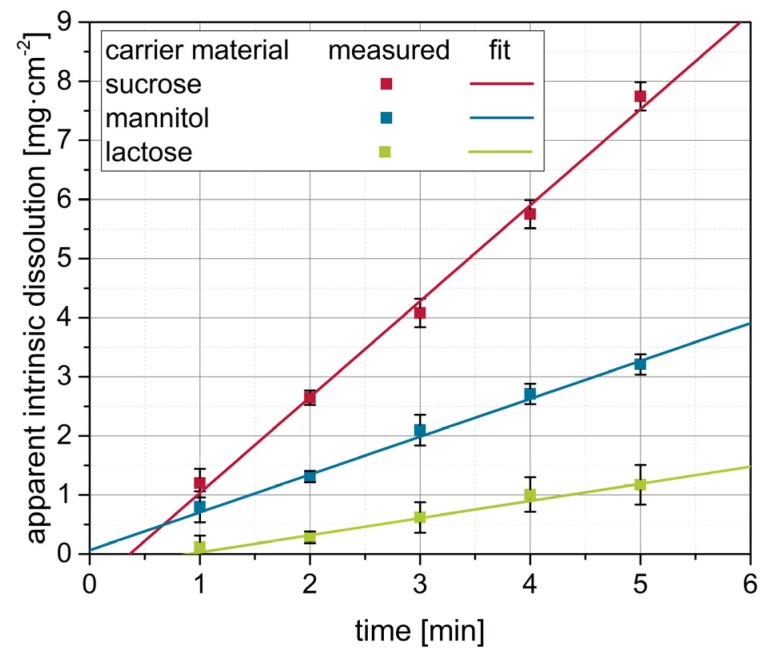
Apparent intrinsic dissolution as a function of time for the different carrier materials determined according to Ph. Eur. 2.9.29 (with modifications, *n* = 3). By linear fitting of the data, the apparent intrinsic dissolution rates of the carrier materials were derived as the slope of the regression line.

Different degrees of nanoparticle embedding can be observed on the surfaces of the granules. The degree of embedding is lowest for lactose, showing a rather rough and porous surface with single naproxen nanoparticles detectable. Nanoparticle embedding increases for mannitol and even more so for sucrose, as indicated by smoother surfaces. As the degree of nanoparticle embedding on the surface of the granules qualitatively correlates with the redispersibility of the corresponding granules (Figure 2), an embedding of the nanoparticles in a protective matrix appears crucial for preventing nanoparticle agglomeration during the drying process and in the final dry state. As only the carrier material was varied during these investigations and all further formulation and process parameters were kept constant during granulation, the differences in redispersibility and nanoparticle embedding within the surface of the granules can be assumed to be caused by properties of the carrier particles. Based on the SEM data obtained and its correlation with redispersibility results, our hypothesis is that partial dissolution of the carrier material occurs at the particle surface upon droplet impingement and that subsequent resolidification of the dissolved fractions causes embedding and separation of the API particles. Would this hypothesis hold, differences in the dissolution rates of the different carrier materials should correlate to the differences in nanoparticle embedding and finally redispersibility. As a fluidized bed granulation process is a dynamic process of wetting of the fluidized carrier particles and drying of the granulation liquid, contact between a single droplet and carrier particle, as well as resulting dissolution of the surface, are assumed to occur in a short period of time. Therefore, one would expect that the dissolution rate of the carrier needs to be high in order to result in effective nanoparticle embedding and separation. Apparent IDRs of the carrier materials were determined based on the procedure of Ph. Eur. 2.9.29 [38]. Results are depicted in Figure 4. These were linearly fitted in order to extract the apparent IDR, i.e., the amount of material that dissolves per unit of area and time.

Generally, it can be seen that the dissolution curves of the carrier materials can be well-fitted (R^2^ > 0.95), and the resulting apparent IDRs increased in the order lactose < mannitol < sucrose (Table 5).

**Figure 5:**
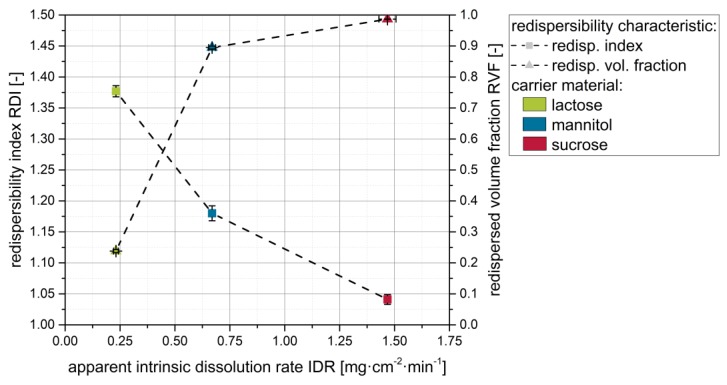
Effect of the apparent intrinsic dissolution rate (IDR) of the carrier material on the redispersibility (i.e., RDI and RVF) of granules produced with a naproxen nanosuspension (stabilized with PVP/VA) without additional drying excipient and different carrier materials (lactose, mannitol, sucrose).

Plotting results from the redispersibility studies (i.e., RDI and RVF) for the different carrier materials against the corresponding apparent IDR indicates that the redispersibility of the granules actually improves with increasing apparent IDR of the carrier materials (Figure 5). This is seen as a proof for our hypothesis that faster dissolution of the carrier particle surface material upon droplet impingement, as indicated by the apparent IDR values, results in more extensive API particle embedding in the carrier material upon resolidification, finally yielding superior redispersibility of the nanoparticles.

The fact that carrier materials with higher dissolution rates lead to a higher redispersibility of nanosuspensions dried in a fluidized granulation process was already observed by Bose et al. [35]. In that study, investigations were conducted with mannitol and lactose, and effects on the dissolution rate of a poorly soluble API were compared. The authors related their investigations to a specific interaction between the carrier particles and the API and/or errors during analytical investigations and did not further discuss or investigate their findings [35]. However, a higher solubility of excipients was observed by Jaiyeoba and Spring to result in coarser and stronger granules during wet granulation processes. This observation was related to a dissolution and subsequent resolidification of the corresponding excipients during granulation, resulting in an increased number of solid bridges between the corresponding particles [39]. These observations are in line with the experimental findings and hypothesis of our own study mentioned above.

A potential alternative cause for differences in redispersibility found in the literature is the size of the carrier material. Azad et al. observed higher dissolution rates and better redispersibility of API nanosuspensions applied to smaller lactose carrier particles in a fluidized granulation process. The authors attributed these findings to differences in the thickness of the API-loaded films around the carrier particles caused by differences in the specific surface area of the different lactose grades. Although no further discussion of how this film thickness may influence the redispersibility of the nanoparticle-loaded granules was provided [32], it may be assumed that the carrier particle size determines the total accessible contact area between the droplets of the API nanosuspension and the carrier material, changing the amounts of carrier material that dissolve and subsequently resolidify during the granulation process. As such, smaller carrier particles with a higher specific surface area might cause a higher degree of nanoparticle embedding and separation, resulting in a better redispersibility. Although we tried to adjust particle sizes by choice of materials and additional air jet sieving of sucrose in the present study, there were still differences in the mean particle sizes (ΔX_50,max_ = 27.0 µm, see Table 1 in Section 2.1) and, consequently, specific surface area of the carrier materials. However, as no systematic dependence of the redispersibility on the particle size and/or specific surface area of the carrier materials was found, the findings of Azad et al. cannot directly be confirmed. This might be attributed to the fact that the difference in particle size was much higher (ΔX_50,max_ = 293.4 µm) in the corresponding study [32]. Whether and how both investigated phenomena (influence of particle size/specific surface area and influence of the dissolution rate of the carrier material) superimpose demand further investigations.

**Figure 6:**
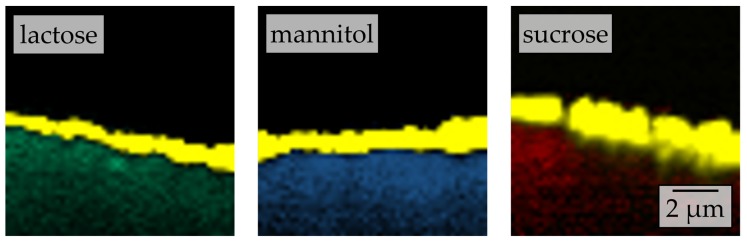
Depth scans conducted by confocal Raman microscopy of granules produced with a naproxen nanosuspension (stabilized with PVP/VA) without additional drying excipient and different carriers. False color representation of reference spectra corresponding to naproxen (yellow), lactose (green), mannitol (blue), and sucrose (red).

As it can be assumed that differences in surface material dissolution and subsequent resolidification during granulation will also result in differences in the thickness of the nanoparticle-loaded layer around the carrier particles, two-dimensional (x-z) chemical mapping depth scans of the granules surfaces were recorded by confocal Raman microscopy (Figure 6).

Clearly, the thicknesses of the nanoparticle-containing layers differ for the different carrier materials in the order lactose < mannitol < sucrose. As stated above, this may be attributed to differences in specific surface area of the carrier materials. However, as the specific surface area of the carrier materials was in the same order of magnitude (0.248–0.266 m^2^·g^−1^), and the rank order of the layer thickness is the same as that seen for the extent of embedding (qualitatively assessed by SEM) as well as that of redispersibility, our hypothesis is seen as further supported.

#### 3.2.2. Influence of Polymer Concentration in the Nanosuspension

**Figure 7:**
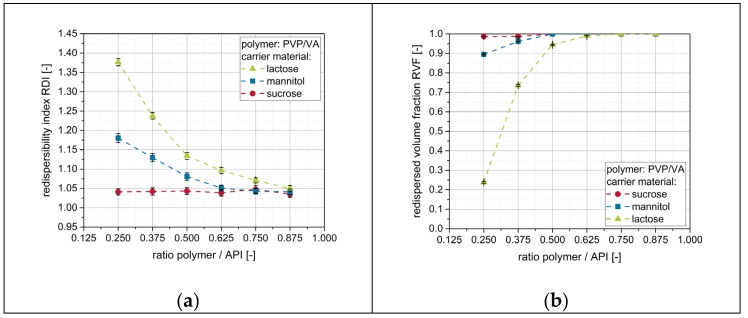
Effect of additional polymer (PVP/VA) in the granulation fluid on the redispersibility of granules produced with different carrier materials (lactose, mannitol, sucrose). Measurement of redispersed suspensions was carried out by means of (**a**) DLS and (**b**) LD.

As nanoparticle embedding on the surface of the granules seems to be crucial for the redispersion of naproxen nanoparticles, additional amounts of polymeric drying excipient (i.e., PVP/VA) were added to the nanosuspensions prior to drying in order to enhance the redispersibility. Results of the corresponding redispersibility studies are depicted in Figure 7.

For mannitol and lactose, for which no complete redispersibility was achieved after applying a pure nanosuspension, the redispersibility improved with increasing amount of additional polymer in the nanosuspensions (i.e., ratio polymer/API). For sucrose as a carrier material, granules were completely redispersible irrespective of the amount of additional polymer in the granulation liquid. Furthermore, data clarify that the amount of additional polymer in the nanosuspension required to obtain complete redispersibility is dependent on the applied carrier material and correlates with the redispersibility when a nanosuspension without additional polymeric drying excipient/matrix material is applied. While both datasets point towards the same trends, slightly different points of ‘complete redispersibility’ can be identified for the different carrier materials depending on the technique used. Whereas results obtained with DLS indicate that granules of mannitol and lactose were completely redispersible for polymer-to-API ratios of 0.625 and 0.875, respectively, data obtained by LD suggest the corresponding granules to be completely redispersible at lower polymer-to-API ratios of 0.500 (mannitol) and 0.750 (lactose). This indicates that a complete redispersion into a nanosuspension (as indicated by an RVF of 1) does not necessarily exclude an increase of the particle size of naproxen during drying (e.g., by agglomeration of single nanoparticles). However, in the current study, an RDI below 1.10 obtained from DLS measurements was always in accordance with a complete redispersion to a nanosuspension, as indicated by data obtained from LD measurements. The corresponding size increase of 10% of the naproxen particles upon drying is regarded as acceptable. Nevertheless, results emphasize the necessity of using both measurement techniques in order to prevent false conclusions regarding the redispersibility of dried nanosuspensions.

**Figure 8:**
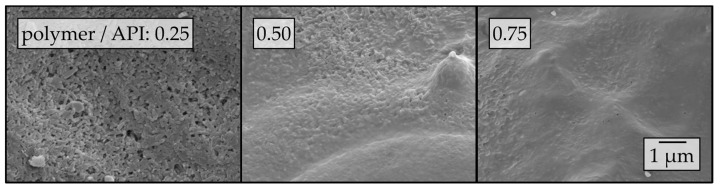
Surface morphologies of granules produced by applying naproxen nanosuspensions with different amounts of additional polymer (i.e., PVP/VA) to lactose carrier particles in a fluidized granulation process, assessed by SEM.

For visualization of the influence of the additional polymer in the nanosuspension, SEM images of surfaces of granules produced with lactose as a carrier material and nanosuspensions with different amounts of additional polymer (i.e., PVP/VA) are presented in Figure 8.

The degree of nanoparticle embedding on the surface of the granules increased with increasing amounts of additional polymer in the nanosuspension. As the redispersibility of the corresponding granules also improved with increasing amounts of polymer, the hypothesis that an embedding of the nanoparticles in a matrix prevents agglomeration during drying and facilitates redispersion can be confirmed. The fact that increasing drying excipient to API ratios improves redispersibility for granules obtained in a fluidized bed process was also observed by Azad et al. [32]. However, they did not attempt to correlate redispersibility to the morphology of the corresponding dried products. Although Melzig et al. investigated a different process by spray-drying nanosuspensions of amorphous ibuprofen nanoparticles, results obtained have relevance to the current study. The authors observed insufficient redispersibility of samples when nanoparticles were not completely embedded within a matrix and related this to an insufficient nanoparticle distance in the granules resulting in increased interactions between the nanoparticles [7]. This argumentation is relevant to the current study and will be further discussed in Section 4.

#### 3.2.3. Influence of Polymer Type in the Nanosuspension

**Figure 9:**
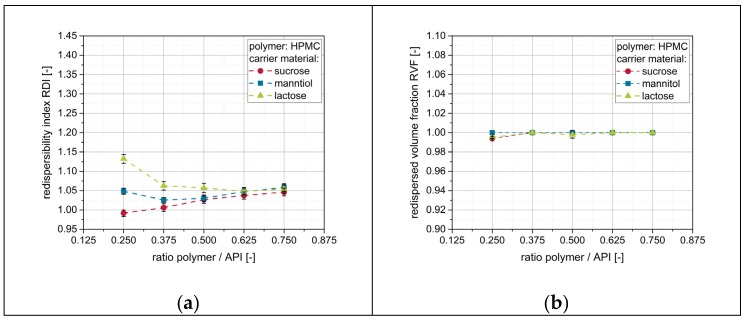
Effect of additional polymer (HPMC) in the granulation fluid on the redispersibility of HPMC/SDS-stabilized naproxen nanoparticles from granules produced with different carrier materials (lactose, mannitol, sucrose). Measurements of redispersed suspensions were carried out by means of (**a**) DLS and (**b**) LD.

Finally, the influence of the applied polymer in the nanosuspension on redispersibility was a further point of interest. Therefore, a naproxen nanosuspension stabilized with HPMC instead of PVP/VA was prepared and sprayed onto different fluidized carrier particles (i.e., lactose, mannitol, sucrose) at different polymer-to-API ratios. An influence of the particle size of the naproxen can be excluded, since the formulations with the two different stabilizer regimes yielded nanosuspensions with comparable particle size distributions, as discussed in Section 3.1 (Table 4). Results from the corresponding redispersibility studies are depicted in Figure 9.

Generally, it can be seen that redispersibility of the obtained granules is good. For mannitol and sucrose as carrier materials, granules were completely redispersible irrespective of the amount of additional polymer in the granulation liquid. However, the particle size (i.e., z-avg.) of the redispersed suspension seems to slightly increase with increasing amounts of polymer in the nanosuspension. This may be attributed to a higher viscosity of the corresponding redispersed samples, as the sample viscosity was observed to clearly influence calculated particles sizes during DLS measurements [40], but the individual sample viscosity was not considered in the corresponding measurement protocol. The fact that this size increase with increasing polymer concentrations was not observed when PVP/VA was applied may be attributed to the fact that HPMC yields higher viscosities than vinylpyrrolidones in comparable concentrations [41]. For lactose, application of a nanosuspension without additional polymeric drying excipient (i.e., polymer-to-API ratio of 0.25) resulted in a slightly compromised particle size of the redispersed suspension, as indicated by an RDI of 1.13. However, complete redispersibility could be achieved with only minor amounts of additional polymer. In comparison with the nanosuspension stabilized with the PVP/VA copolymer, application of HPMC as a stabilizer in the nanosuspension resulted in a better redispersibility of the granules for all carrier materials at a given polymer concentration. Consequently, polymer consumption to achieve complete redispersibility can be reduced by the factor of up to 2 when HPMC is used as a polymeric drying excipient instead of PVP/VA.

**Figure 10:**
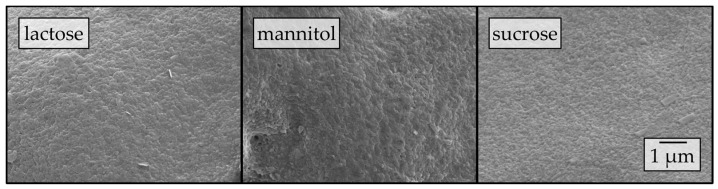
Surface morphologies of granules produced with a nanosuspension (stabilized with HPMC) without additional drying excipients and with the use of lactose, mannitol, and sucrose as carrier materials in a fluidized granulation approach, assessed by SEM.

Surface morphology of granules produced with a pure nanosuspension stabilized by HPMC (Figure 10) reveals high degrees of nanoparticle embedding within the surface of the granules, irrespective of the applied carrier particle type. Consequently, a high degree of nanoparticle embedding qualitatively correlates with a good redispersibility, as seen in previous sections.

An interesting resulting question is why the application of HPMC causes a higher degree of particle embedding as compared to PVP/VA. It may, for example, be assumed that the application of HPMC causes a higher viscosity of the nanosuspensions, resulting in larger droplet sizes at a given atomization air pressure [42], which was observed to greatly influence the outcome of a fluidized bed granulation process [43]. A larger droplet size in turn may affect the degree of carrier particle dissolution and subsequent resolidification, described in Section 3.2.1, by extended contact times between droplets of the nanosuspension and the carrier particle surface. In addition, it cannot be excluded that other properties of the polymers or specific interactions between the polymers and the API influence the outcome of redispersibility studies and/or the degree of nanoparticle embedding on the surface of the product granules. However, for clarification, further investigations are indispensable.

**Figure 11:**
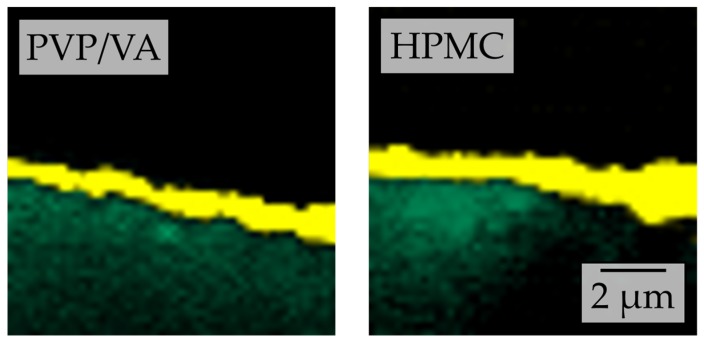
Comparative depth scans conducted by confocal Raman microscopy of granules produced with a naproxen (yellow false color according to reference spectra) nanosuspension stabilized with PVP/VA or HPMC without additional drying excipient and with lactose (green false color according to reference spectra) carrier particles.

Nevertheless, comparative Raman chemical maps of granules produced with nanosuspensions without additional polymeric drying excipient (i.e., polymer-to-API ratio of 0.25) and lactose as carrier material were generated for both stabilizer regimes (Figure 11). These indicate that HPMC qualitatively causes a thicker API-containing layer around the carrier particles than PVP/VA at a given concentration. As the same amounts of naproxen are applied to the same carrier material and just the polymer in the nanosuspension differs, this again points to the nanoparticles being distributed in a larger volume of drying excipient/matrix material. As a result, the mean distance between the naproxen particles can be assumed to be longer, resulting in a decreased interaction between individual naproxen particles and yielding better redispersibility for HPMC [7,40].

## 4. Conclusions

Preservation of redispersibility is a key objective during the conversion of API nanosuspensions into the solid state in order to maintain the advantageous properties of the nanoparticles with regard to bioavailability of the API. The results of this study underline the importance of adapting formulation parameters during application of a naproxen nanosuspension in a fluidized bed process in order to prevent agglomeration of nanoparticles. Furthermore, irrespective of the applied formulation, the redispersibility of the product granules was observed to qualitatively correlate with the degree of nanoparticle embedding within the surface of the corresponding granules. Additionally, Raman chemical maps qualitatively revealed differences in the thickness of nanoparticle-loaded layers around the carrier particles for selected formulation parameters (i.e., carrier particle type, type of polymer in the nanosuspension). The dissolution rate of the carrier material and the type of the polymer were identified as the determining factors for layer thickness. Irrespective of the cause for observed differences in the degree of nanoparticle embedding, this embedding can be directly attributed to the dispersion of the naproxen nanoparticles in a carrier/polymer matrix and finally to different distances between individual nanoparticles. According to Melzig et al. [7], an insufficient separation of nanoparticles and, consequently, low distances between individual particles may result in higher potential of interaction, finally causing nanoparticle agglomeration and a poor redispersibility. On the other hand, better separation of the particles and corresponding larger distances between individual nanoparticles may prevent an agglomeration and result in a preservation of redispersibility. Consequently, it may be assumed that the redispersibility of a dried nanosuspension is determined by the distances between the API nanoparticles in the dry state. However, while this study rather gives a qualitative correlation between the redispersibility of the products and the dispersion of the naproxen nanoparticles in a carrier/polymer matrix, a theoretical or exemplary description of these relationships, following the example of Steiner et al. [40], may enable a prediction of the formulation design for producing API nanoparticle-loaded granules with predefined properties. In this regard, the results presented in this study identify crucial formulation parameters during drying of naproxen nanosuspensions in a fluidized bed process and hint to the underlying mechanism for the preservation of redispersibility of the corresponding products

## Figures and Tables

**Table 1:** Characteristics of particle size distributions and specific surface areas *S_m_* (both derived from laser diffraction measurements), as well as solid densities *ρ_solid_* (determined by gas pycnometry) of carrier materials used for fluidized bed granulation.

Carrier Material	X_10_	X_50_	X_90_	S_m_	*ρ_solid_*
	[µm]	[µm]	[µm]	[m^2^·g^−1^]	[g·cm^−3^]
lactose	14.6	57.7	139.0	0.266	1.5366
mannitol	10.4	71.5	238.0	0.263	1.4839
sucrose (air jet sieved)	16.2	44.5	111.0	0.248	1.5852

**Table 2:** Polymer to active pharmaceutical ingredient (API) ratios and resulting mass fractions of carrier material, polymer, and API in the granules.

Ratio Polymer/API [-]	*c_m,carrier_*^1^ [-]	*c_m,polymer_*^1^ [-]	*c_m,API_*^1^ [-]
0.250	0.889	0.022	0.089
0.375	0.878	0.033	0.089
0.500	0.867	0.044	0.089
0.625	0.856	0.055	0.089
0.750	0.844	0.067	0.089
0.875 ^2^	0.833	0.078	0.089

^1^ SDS was neglected; ^2^ only for PVP/VA.

**Table 3:** Porosity of carrier compacts (and corresponding standard deviations (n = 3)) used for the determination on the apparent intrinsic dissolution rate of carrier materials according to Ph. Eur. 2.9.29.

Carrier Material	ε_compact_ [-]
sucrose	0.118 ± 0.004
mannitol	0.113 ± 0.002
lactose	0.120 ± 0.008

**Table 4:** Particle size (i.e., z-average) and PdI of the naproxen nanosuspensions directly after milling and after storage at ambient conditions.

Stabilizers	After Milling		3 Days of Storage		7 Days of Storage	
	z-avg.	PdI	z-avg.	PdI	z-avg.	PdI
	[nm]	[-]	[nm]	[-]	[nm]	[-]
2.50 wt% PVP/VA + 0.25 wt% SDS	132.7	0.13	147.5	0.13	154.1	0.11
2.50 wt% HPMC + 0.25 wt% SDS	141.6	0.12	153.2	0.14	155.9	0.14

**Table 5:** Apparent intrinsic dissolution rates (IDRs), corresponding standard deviations and R^2^ of linear fits of intrinsic dissolution data determined according to Ph. Eur. 2.9.29 (with modifications) for the different carrier materials (*n* = 3).

Carrier Material	Apparent IDR [mg·cm^−2^·min^−1^]	R^2^ [-]
sucrose	1.623 ± 0.062	0.994
mannitol	0.640 ± 0.029	0.992
lactose	0.290 ± 0.031	0.956
